# Fourteen Main Obstacles on the Journey to Post-Traumatic Growth as Experienced by Female Survivors of Intimate Partner Violence: “*It Was All So Confusing*”

**DOI:** 10.3390/ijerph19095377

**Published:** 2022-04-28

**Authors:** Hulda S. Bryngeirsdottir, Sigridur Halldorsdottir

**Affiliations:** School of Health Sciences, University of Akureyri, Solborg v/Nordurslod, 600 Akureyri, Iceland; sigridur@unak.is

**Keywords:** post-traumatic growth (PTG), intimate partner violence (IPV), gender-based violence (GBV), trauma recovery, public health, rehabilitation, women’s health, interdisciplinary approach, phenomenology, qualitative research

## Abstract

In this study, we identified 14 obstacles experienced by female survivors of intimate partner violence who had, nonetheless, reached post-traumatic growth (PTG), which is a positive psychological change by a person following serious difficulties or traumatic events. Intimate partner violence (IPV) is such a trauma. The purpose of this study was to analyze the obstacles to PTG as experienced by women who have succeeded in reaching PTG following traumatic IPV. Participants were twenty-two women aged 23–56 who self-reported their PTG according to the working definition used. The participants reported feelings of diminished self-worth that had negatively influenced their lives and how these negative feelings delayed their PTG. The overriding theme of the study was “It was all so confusing”, which expressed the essence of the participants’ feelings when describing the obstacles they encountered on their journey to PTG. Most of those obstacles were intrapersonal, i.e., negative personal feelings and negative perspectives towards themselves. Other obstacles reported by participants were physical and psychological health problems, challenging personal circumstances, and the perpetrator, as well as laws, regulations, and institutional social systems. This study reveals the broad range of obstacles encountered by women on their journey to PTG following IPV, emphasizing the necessity of an interdisciplinary approach when holistically considering their situation and supporting them on their journey towards PTG.

## 1. Introduction

When facing the broad range of diverse debilitating effects of gender-based violence, most survivors demonstrate a remarkable capability for survival and endurance [1,2]. Leaving an abusive relationship results in transformative changes in life for survivors of IPV, as they move from being controlled to being in control of their own lives [3,4]. Even though research on the aftermath of IPV has mainly focused on the adverse consequences of that experience [5], the awareness of the possibility of positive changes following IPV has risen, where the strength, resilience, and other positive resources of survivors of IPV are realized and emphasized [6,7,8]. Some women can recover from their experience of IPV, but there is a lack of information on how they recover and whether the recovery is for the long term [7,9].

Gender-based violence (GBV) is an extensive and serious social problem globally, affecting approximately one in three women, regardless of their social circumstances or ethnicity. Domestic violence is the most common kind of GBV [5,10]. According to the UN Declaration on the Elimination of Violence against Women, GBV is defined as “any act of gender-based violence that results in, or is likely to result in, physical, sexual or psychological harm or suffering to women, including threats of such acts, coercion or arbitrary deprivation of liberty, whether occurring in public or in private life” [9,11]. Victims of GBV are at an increased risk of having serious physical, mental, sexual, and reproductive health problems. They are also at an increased risk of being murdered by their intimate partner [10]. 

Intimate partner violence (IPV) is one of the most common types of violence against women [10]. The definition of IPV is any behavior in an intimate relationship that causes the victim of the violence physical, psychological, or sexual harm [10,12,13]. IPV also refers to controlling behaviors such as isolating the spouse from other people, monitoring their actions, and dominating and restricting their finances, education, occupation, and health care [12,13]. Globally, research has shown that women are more likely to be assaulted, injured, raped, or killed by a current or former spouse than by anyone else, and the perpetrator is most often a male [14,15]. Psychological aggression is the most common type of IPV. That kind of violence in intimate partner relationships is likely to have a long-term devastating effect on the psychological health of the victim, resulting in depression, anxiety, post-traumatic stress disorder (PTSD), and reduced self-kindness, thus leading to less positive reframing of stressful events in life, less meaning in life, and less growth and maturity [16], as well as a loss of internal ego structure among the victims [17]. IPV has been described as “intimate terrorism”, where the perpetrator is in control, abusing the partner emotionally using threats, intimidation, economic abuse, and guilt [18,19]. The dynamic in violent relationships has been described by Dr. Evan Stark as “coercive control”, which captures the multidimensional nature of IPV, wherein psychological violence plays a large role in the controlling and intimidating behavior of the perpetrator, along with the social isolation of the victim [20]. To leave an IPV relationship is a long-term, complicated process, even after the end of the violent relationship [21,22,23], and it also takes tremendous strength to start a new life after leaving a violent relationship [1]. Besides the negative physical and mental outcomes of IPV, such an experience often includes adverse economic consequences, housing instability, and social stigma [24]. The process of help-seeking after experiencing IPV appears to be very complex due to various individual and social factors [25,26].

Post-traumatic growth (PTG) is a positive psychological change by a person following serious difficulties and traumatic events where the focus is on the possible positive outcomes of experiencing trauma rather than focusing on the negative outcomes [27]. According to the definition of PTG by Calhoun and Tedeschi [28] and Tedeschi and Moore [29], the person discovers new opportunities in life, values life more, enjoys increased spiritual maturity, experiences increased personal strength, and has better relationships with others. PTG has been described as a journey, where one’s internal factors and need for change are the motivation for PTG [8]. According to the theoretical definition of PTG by Bryngeirsdottir and Halldorsdottir, PTG is explained as a personal resurrection in life following psychological trauma, including the person confronting their own feelings, experiencing intensified inner strength, having deeper relations to others, experiencing personal growth, living a healthier life, enjoying increased self-knowledge as well as a stronger self-image. The authors state:

“Furthermore, the individual enjoys increased social activity, positivity and patience and has feelings of freedom, power, and energy, without any regrets. Moreover, the individual feels like a winner in life, is less stressed, more appreciative of own self, others, and life in general, seeing new possibilities in life having found a new vision as well as deeper inner peace and wisdom. Even though the negative influences of trauma can be present, the positive factors of post-traumatic growth are dominant”.

[8] (p. 13)

Experiencing IPV is likely to result in extensive, long-term devastating effects on the survivors’ health and her life as a whole. Furthermore, IPV is likely to also affect her friends and family as well as her community in a negative way. That said, aiming for PTG following IPV in order to enjoy the best life possible, is not only in favor of the survivor of IPV but also her loved ones and her society as whole.

In a study of women who self-reported their PTG following IPV, participants expressed their perception of PTG as a “personal, inner reconstruction of themselves”, where they emphasized the intrapersonal concepts that described their PTG, focusing less on the interpersonal factors [30] (p. 15). Discovering and addressing the hindrances and the facilitating factors on the journey to PTG following the experience of IPV is essential in order to support female survivors of IPV on their journey to PTG and better lives.

The participants of this study were Icelandic female survivors of IPV. According to a national survey in Iceland since 2020, where the prevalence of hospital visits and nature of injuries because of IPV against women and associated costs were analyzed, the lifetime prevalence of sexual and/or physical IPV against women in Iceland was at 22.4% and the current prevalence at 1.6% [31]. An older study from 2010 showed similar results, the prevalence of sexual and/or physical IPV being 22% [32]. These Icelandic results are comparable to results on the subject from one of the largest face-to-face interview studies ever conducted with 42,000 women, on women’s experiences of violence in 28 European countries, where the results showed that 22% of participants had experienced physical and/or sexual violence in an intimate relationship [33]. The authors did not find any statistics regarding the prevalence of other forms of IPV among Icelandic survivors of IPV. Overall, there seems to be severe lack of information regarding PTG among survivors of IPV, in Iceland.

### 1.1. Rational for Conducting the Study

IPV is of multidimensional nature and so is the trauma recovery that follows. The results of IPV are well documented, while the possibilities of PTG following such trauma have not had much attention in the literature, though the attention to possible PTG following IPV is growing. Finding and analyzing the obstacles to PTG among female survivors of IPV is essential in order to be able to support women when working their way to PTG following IPV.

### 1.2. Purpose of the Study and Research Question

This study aimed to explore the obstacles on the journey to PTG as experienced by female survivors of IPV. The phenomenon was studied from the perspective of women who succeeded in reaching PTG following IPV. The main research question was: “*What are the main obstacles on the journey to post-traumatic growth as experienced by Icelandic female survivors of intimate partner violence?*”

## 2. Materials and Methods

### 2.1. Study Design

To answer the research question, the Vancouver School of Doing Phenomenology (Vancouver School) methodology was used. This research method aims to increase the understanding of the participant’s experience of a particular human phenomenon and is a combination of the works of Spiegelberg [34] on phenomenology, Ricoeur’s [35,36] hermeneutic phenomenology, and Schwandt’s [37] constructivism. The Vancouver School is popular among nurse researchers in the Nordic countries because of its 12-step approach, which has been found clear and useful [38]. It has been proven to be an excellent research methodology when the participants belong to a vulnerable population. When using this research method, participants report their experience of a certain phenomenon, and researchers try to comprehend and describe their experience, with the purpose of advancing human services through expanding knowledge and understanding of human phenomena [39].

### 2.2. Participants

Purposeful sampling was used in this study and participation was anonymous and voluntary. One criterion for participation was having reached PTG following IPV. Participants self-reported their PTG, according to the working definition used in this study (see Table 1).

The criteria for participation in this study also included the participants being at least 18 years old, being able to understand and read Icelandic, and status of at least one year since their violent relationship ended. 

### 2.3. Data Collection and Analysis

Participants in this study were recruited by introducing the research in numerous ways. During the recruitment the researchers promoted the research, e.g., by verbally introducing it in various places, introducing it online, using social media and various webpages, by handing out flyers, and sending e-mails to groups of different women’s associations. Thirty-four female survivors of IPV, who self-reported their experience of PTG (based on the working definition of PTG, see Table 1), signed up for participation in the study. However, before the interviewing began, the COVID-19 pandemic started, making face-to-face interviewing impossible. When face-to-face interviewing was considered safe again, twenty-two women, aged 23–56, who still wanted to participate in the study were interviewed. The data collection and the data analysis were conducted by following the 12 basic research steps of the Vancouver School (see Table 2).

**Figure 1:**
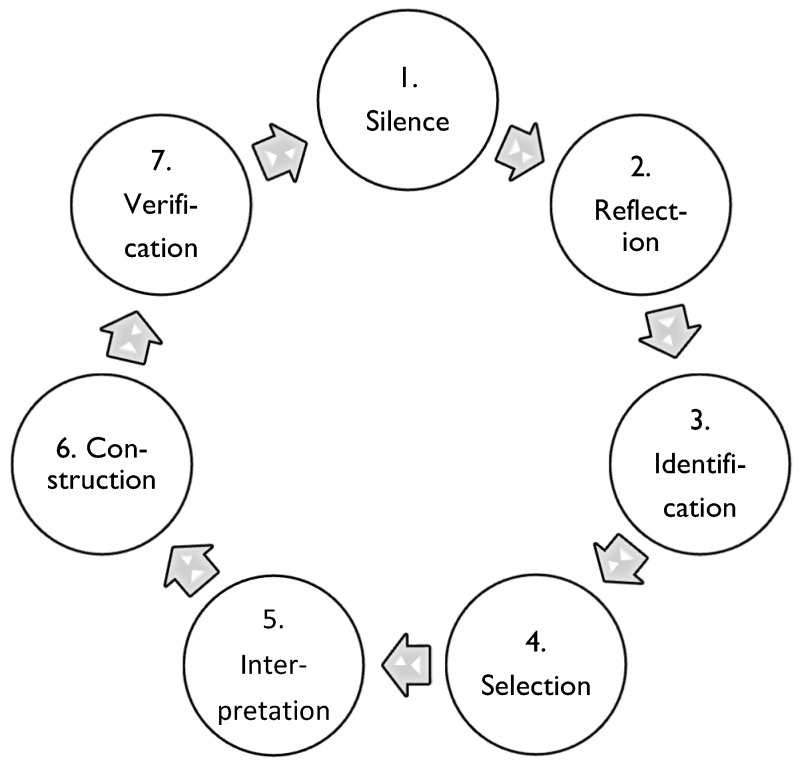
The research process of phenomenology in the Vancouver School ([39], p. 56). Reprinted with permission from Ref. [39]. 2000, Sigridur Halldorsdottir. This cycle is repeated in every step of the research process of the Vancouver School method.

An overview of the research process followed in each step of the Vancouver School method (see Table 2) can be found in Figure 1.

#### 2.3.1. Data Collection

The researchers developed an interview guide based on the research question and the literature that was followed during the semi-structured interviews. Each participant was interviewed once by the first author, in a neutral, quiet, and safe place chosen by the researcher, with no one else present. Since very little is known about the obstacles on survivors’ journey to PTG following IPV, the authors wanted to study all aspects of it, as well as the nuances of the experience in detail; therefore, 22 interviews were conducted.

All the interviews were recorded, transcribed verbatim on a computer, and encrypted, their duration being 39–134 min (M = 77 min). Before the beginning of each interview, the first author introduced the main interview question and the working definition of PTG, and explained the purpose of conducting the research. Each participant confirmed her understanding and signed an informed consent. The first author encouraged each participant to express herself freely and openly during the interview. At the end of each interview, the participant was given the name of a professional (a psychologist and a psychiatric nurse) who she could contact and have a free therapeutic session if she felt she needed that after disclosing her experience of the obstacles to PTG following IPV. 

The main interview question and examples of follow-up questions from the semi-structured interview guide used in this study are shown in Table 3. 

#### 2.3.2. Data Analysis

The first author implemented preliminary data analysis under the supervision of the second author. The first author repeatedly read the transcripts and analyzed them in detail by marking texts and writing comments in the margins, which contributed to answering the research question. NVivo 12 was also used for the same purpose during the data analysis. Every interview was further analyzed through coding, categorizing, and organizing the data into main themes and subthemes to construct the essence of the participants’ experience, using the research process of the Vancouver School (see Figure 1). The main themes and subthemes in each woman’s story were emphasized and the most significant themes were built into an individual analytic framework. For verification, all the interviews were re-read and compared to the final analytic framework, resulting in the following overriding theme, describing the phenomenon: “It was all so confusing”, which expresses the core of the participants’ feelings when describing the obstacles on their journey towards PTG. When writing the findings, the participants were quoted directly to increase the credibility and the trustworthiness of the findings and conclusions. 

### 2.4. Research Ethics

The researchers in this study were guided by the fundamentals of research ethics. The National Bioethics Committee authorized the study (reference concealed for review). All the participants received an introductory letter, where they were informed about the purpose of the study, the research method, and what their participation entailed. They were informed of their right to participate voluntarily and to withdraw from the study whenever they wanted, as well as the anonymity and confidentiality of their participation. This information was repeated before the beginning of each interview, to ensure the understanding of the woman and her willingness and consent to participate in the research. Throughout the study the researchers emphasized deidentifying and minimizing the risk of harm to the participants, by recording each interview and transcribe it verbatim. All interviews were kept in a locked area. Because of the vulnerability of the participants, anonymity and confidentiality to the women was emphasized. To further ensure the rights and welfare of the participants following the interviews, all of them were offered psychological support from a professional without charge if they felt they needed it, without any of them using that offer. 

### 2.5. Validity and Reliability

The motivation for conducting this research comes from the first author’s work as a vocational rehabilitation counselor in Iceland, and from her work with women living in a halfway home for women and children following IPV, which is run by the Icelandic Women’s Shelter in Iceland. The second author is a professor with extensive experience in qualitative research, mostly around violence and psychological trauma. The authors have conducted prior research on PTG with Icelandic people, suffering various types of traumas, resulting in a new theoretical definition of PTG [8]. When combining those research results and the first authors’ experience of working with women following IPV, the authors decided to conduct this research. The authors find it not acceptable, that a woman who has been violated against by her intimate partner endures long time suffering due to that experience, without a hope of possible positive changes in her life. All this resulted in the authors’ interest in doing research on identifying the obstacles on their journey to PTG. 

The authors were aware of the importance of reflexivity in the research process of this study, especially due to their prior experiences explained above. The research process of the Vancouver School is designed to increase validity and reliability, where the researchers constantly reflect on their preconceived ideas of the phenomenon and consciously put them aside as much as possible. Thus, the research method used in this study is suitable in the attempt to prevent bias impacting the results, particularly when the researchers are connected to the phenomenon being explored as explained above.

Data saturation was reached when the data was sufficiently dense and both authors agreed that the research question could be answered.

## 3. Results

The overriding theme of the study was “It was all so confusing”, which describes the participants’ essential experience of the obstacles on their path to post-traumatic growth following intimate partner violence, together with the immense destructive effects that the violent relationship had on their lives. The participants experienced various obstacles on their journey to PTG that resulted in a delay of their PTG following their experience of IPV. The women explained how hard it was for them to confront and process their own negative, personal feelings towards their experience of violence and how their negative attitude and feelings reduced their chances of achieving PTG. They felt distressed, and many of them reported difficulties in starting to process their experience of IPV. The participants’ psychological and physical health was not good in general, and many of them described their overall situation as being difficult, which influenced their path to PTG in a negative manner. Their personal circumstances and social surroundings were demanding, and many participants experienced a lack of support and felt like they were being judged at the time, resulting in decreased possibilities of PTG. Many of the perpetrators continued their harassing behavior, and when it came to supporting the women in reconstructing and continuing with their lives or breaking their connection to the perpetrator, the law and institutional social systems were often not helpful. 

There were fourteen main obstacles identified in the women’s accounts. These obstacles tended to keep the women in a survival mode, which kept them from processing their immensely negative experience to recover, to heal, and to reach PTG. Our findings regarding the fourteen obstacles on the journey to PTG as experienced by female survivors of IPV are shown in Table 4.

The women experienced various negative personal feelings following their experiences of violent intimate relationships, which had immense destructive effects on their lives. They described how hard it was for them to confront these negative feelings and how afraid they were of starting to talk about their experience of IPV, because of their fear of being stigmatized. This resulted in a delay in them starting to process their experience of IPV, which were obstacles on their journey towards PTG.

*When you face yourself, you can break and become vulnerable. I was really late in starting to process my experience of my violent relationship, I was the hindrance in starting this process. I should have started earlier, then possibly I wouldn’t have lost my health*

(Frida)

*At first, I downplayed the violence and took the blame and responsibility of the situation. I did that to survive*

(Ursula)

**Feeling of shame.** The majority of the participants experienced a heavy feeling of shame, both during the violent relationship and after the relationship had ended. Many of them found the feeling of shame the hardest part to endure and it had great negative effects on their lives and wellbeing, which delayed their PTG. They blamed themselves for being in a violent relationship and felt that they should have known better. Some of them said they had been warned in the beginning of their relationship and were ashamed that they did not listen.

*I always thought this relationship could work. It was a great defeat and really hard to face the fact that it didn’t. I had been warned, this was all my fault. Right there I made a great mistake, that contributes to being so ashamed of all this*

(Frida)

Others felt that their social status, job, or education was such that they should have known better. They assumed that they would lose their social and personal credibility if they reported that they had been in an abusive relationship.

*I didn’t picture myself as a woman or a girl who would ever be in such a relationship. That’s why I couldn’t think of it as a violent relationship at the time. And all I felt was just feeling of shame, a shame of being in this situation*

(Iris)

This strong feeling of shame caused them to avoid sharing their experiences with others, thus negatively affecting them in processing their experience of IPV. They feared that by sharing their experience with others, they would lose their credibility and would be stigmatized by their community and by society.

*I only shared some small pieces of my experience. I was so ashamed of it*

(Bella)

*The fear of admitting the whole thing, the violence, it was enormous. I thought I should have known better or something. I am not sure what it was. Enormous shame, the fear of talking about this and to share it with someone in our small community, was so enormous*

(Cecilia)

Some participants described how their feelings of shame, because of their experience of IPV, prevented them from finding appropriate support to start processing their experiences.

*I couldn’t find the best way to start processing this. There was so much shame involved. I was so functional in one part of my life, my work, my study… and so dysfunctional in another part of my life, my private life. I was so ashamed; I couldn’t ask anyone for help*.

(Julia)

In some cases, this feeling of shame influenced the women in a negative way when they started dating again. They felt like their worth was less after being in a violent relationship and were afraid that other people felt the same.

*I felt ashamed and felt like I didn’t belong anymore, that I didn’t have the same worth as other people. That feeling affected me when I tried to start dating again. “What if he knows about me and my experience of violence?”. I really had to process that feeling*

(Wilma)

**Suicidal thoughts.** Many women described their difficulties in finding a purpose in life after their experience of IPV, which delayed their PTG. They blamed themselves for all kinds of things and some of them reported suicidal thoughts after the end of their violent relationship.

*I was standing in the shower, thinking: “I’m always fucking everything up for myself, I will ruin my child’s life and he will end up as fucked up as I am… I really should end this and take my own life.” I thought that everything would be better if I didn’t exist*

(Vanessa)

*After this relationship, I felt like I was the scum of the earth, I just wanted to kill myself*

(Phoebe)

**Broken self-identity.** Participants described their negative attitude and anger towards themselves following their experience of IPV. Their self-identity was broken, and their body image was distorted. They continued to bash themselves, accusing themselves of being inadequate, and criticizing themselves in many destructive ways, resulting in delay of their PTG.

*This thought, it just stuck with me, that I could have left this violent relationship, and since I didn’t, I just got what I deserved, it was all my fault. I didn’t deserve anything good in life*

(Grace)

*Even after the divorce, I never looked at my body in a mirror, because I thought my body was appalling*

(Natalie)

*I was angry with myself, that I had allowed this relationship to go on for such a long time, that I had allowed this to happen*

(Therese)

**Insecurity.** Some women reported that they felt anxious and insecure after their violent relationships had ended. They did not know who they were anymore, felt aimless in life, and did not know what to do next.

*I was in free fall actually; I didn’t know how to behave or know who I was. I had been living in some kind of drama theater, where everybody was supposed to play their role. And then one night it was over. The play had ended*

(Eva)

*My life had become so small, and I had become so secluded, all my life had been built around this one man. It took time to enlarge my life in a secure way, it took some time for me to realize that I could fly*

(Ursula)

**Feeling alone and isolated.** Many participants felt alone and lonely, since they had either been isolated by their perpetrator or had isolated themselves during their violent relationship. This feeling of loneliness was uncomfortable and made them even more vulnerable, thus delaying their PTG.

*At the end, somehow, there was no one left there for me, there was no one around anymore*

(Cecilia)

*Somehow, I was all alone after this relationship. I hadn’t yet reconnected with my friends*

(Grace)

**Triggers.** Some participants explained how different events or things could trigger bad feelings related to the violence they had endured. Even though the nature of triggers differed between the participants, all the triggers had negative effects on their wellbeing and needed to be processed. Triggers delayed the women’s’ PTG in many cases.

*When I am driving, years later, and I see a yellow jeep, like he [the perpetrator] used to have, I still feel a bit triggered*

(Julia)

*In my second pregnancy I experienced many triggers, due to my ex-husband’s behavior during my first pregnancy. I had to say to myself that my current spouse would not treat me like my ex-spouse did during my pregnancy. I really had to explain that out loud to myself, multiple times*

(Kimberly)

*There is this man, who sometimes writes horrible things about women in the newspaper. I know what this man has done to other women, and to read the columns he writes, triggers me, I get angry and upset*

(Vanessa)

**Mixed negative feelings.** After the end of the violent relationship, all kinds of feelings came up, such as relief, grief, anger, regret, fear, and vulnerability. Those feelings were difficult and needed to be processed, before the women were able to continue their journey to PTG.

*At first, I felt relieved and embraced the thought of me never going back to this situation, but when I started to think about it in depth, I experienced such a strong feeling of grief. When I finally realized what had happened. I experienced all kinds of feelings*

(Ursula)

*I felt so sad after all this, I experienced so much grief and regret because of my broken dream, the dream about my future happy family that I had wished for, but I didn’t get after all*

(Bella)

*At first, I went through a typical process of grief; denial, anger and all the feelings that are related to grief*

(Yvonne)

*Sometimes I regret all the years that I wasted on this relationship*

(Rachel)

**Emotional connection with others.** Some of the women described problems in trusting and/or connecting emotionally with other people after their experience of IPV. This lack of trust applied to people in general, both men and women, and delayed their PTG.

*I just don’t trust other people anymore, especially not men*

(Phoebe)

They also explained how some innocent use of words by their new spouse, the tone of their voice, or some situations in their new romantic relationship could be triggering for them. Their reaction sometimes could be hard for their current spouses to endure and understand.

*After my experience of IPV, I was always insecure and afraid in romantic relationships. I was always waiting for something bad to happen. So, I was not good in being in relationships after that*

(Vanessa)

*My current husband, who is the sweetest man on Earth, once said some innocent words to me when we had guests, words, that somehow triggered me. After our guests left, I just snapped. I was so angry with him. Just because the words he used reminded me of something bad from my former violent relationship. My poor, adorable husband, he was shocked [laughter]*

(Yvonne)

It could be difficult for the women to enter a new romantic relationship due to their changed or lost social standards and the feeling of shame and stigmatization felt after being a victim of IPV.

*When I entered a house in my old, fancy neighborhood, that belonged to a man I was dating, it came so clear to me. I had an old car, I had no money and I was officially a victim of IPV. I didn’t belong there, we weren’t equals… So I left. I couldn’t date him*

(Wilma)

**Physical and psychological health.** Participants described how their experience of intimate partner violence affected their physical and psychological wellbeing in a negative way, even though they had left the relationship, resulting in a delay of their journey to PTG. They blamed their health problems on the severe and long-term stress they endured in their violent relationships, where they always had to be alert, trying to please the perpetrator in an attempt to prevent him from being violent.

*Around six months after this relationship ended, I got very sick, physically. I ended in a hospital where I was diagnosed with an intestinal disease, some bleedings in my stomach and my intestines, fibromyalgia and more. And mentally, I was just broken my spirit was extremely broken after this relationship (silence). I had a really tough time there*

(Frida)

Many participants talked about being tired all the time following the end of their relationship and some of them had so little energy that they could not work.

*I went to see my doctor, who did some blood tests and more to find out why I was so tired all the time. There was nothing physically wrong with me, my constant fatigue was due to long-term psychological stress*

(Lydia)

*My health got worse after my divorce. And after my divorce I had to take care of everything on my own, the household, the children. After all that had been going on, I didn’t find the energy to do that*

(Phoebe)

Some of the women reported that their former spouse gaslighted them in order to make them do and behave as he pleased, resulting in them not comprehending the severity of the violence until after the relationship had ended. This was one of the reasons why they did not realize their health problems until after the end of the relationship—they did not have enough space to realize it before.

*I didn’t realize how severe the psychological violence had been or how badly it had affected my health until long after our separation. He had gaslighted me for such a long time, and I just agreed because I wanted to have peace in our home*

(Heidi)

Some participants continued to treat themselves in a destructive way after the end of their relationship due to their long experience of violence.

*I was really relieved after I got divorced, but even so, I was psychologically violent and hostile towards myself for many years after the divorce. I’m slowly getting over these feelings towards myself, but I still feel fragile when it comes to this*

(Natalie)

**Personal circumstances and social surroundings**. Participants’ personal circumstances were often difficult after the end of the violent relationship due to their social circumstances, financial status, and other traumatic occurrences that arose in their lives after the violent relationship had ended, delaying their PTG. 

Many women described how their ex-spouse had isolated them and how they themselves had pushed other people away for their own good. Thus, when their relationship finally ended, they felt they were alone.

*When the relationship ended, I realized how isolated we had become. We were isolated from my loved ones, family and friends and people in general. I know they were worried about me, but they had been pushed away, he always gave me trouble when I wanted to see them. So, I didn’t have any security net at first after the divorce*

(Bella)

*I didn’t have any real friends after all the years that I lived with him. I just stayed home, taking care of him and raising the children, and little by little I lost the few friends I had*

(Wilma)

Some described their continuing codependency resulting from their family of origin, their violent relationship, or both, which affected their PTG in a negative way. They had trouble setting boundaries with other people, resulting in bad choices of friends and relationships with others.

*I was so codependent, I was having real trouble in setting boundaries to others, a lot of things had to go on before I said “stop” to someone*

(Frida)

*I was very young when I learned to please other people and if I felt that something was not right, I just learned to suppress my opinion and say nothing*

(Kimberly)

The women blamed themselves and were ashamed of their situation at first. They were afraid of talking about their situation or asking for help because they feared that their loved ones and other people would also blame them and judge them for their circumstances.

*I was always afraid that people would see me differently, see me as a victim or think: “she can blame herself, what was she thinking? Should I feel sorry for her now?” I have never had such negative reaction from anyone*

(Grace)

*I think it’s the same feeling by all people who have suffered violence, you blame yourself, you are afraid that no one will believe you and you are afraid of the reaction you will get. And in some cases, you are right to feel that way. There is still so much prejudice out there*

(Iris)

Some of the participants were not able to work due to health issues following their violent relationship, resulting in them taking sick leave or even facing long-term disability. To be in that situation turned out to be difficult for them; they lost their routine and some of them became socially isolated.

*I was always tired, just worn out. So, I couldn’t work. I have always been able to work and take care of myself so it was very hard for me to face the fact that I couldn’t anymore. I was worried that I would go insane at the time*

(Marianne)

*I have always had a job, I need my routine. I lost my routine, just when I needed it the most, just when I was starting to process my trauma. I realized later that it was good for me, but it was really hard at the time*

(Eva)

Many participants reported great financial difficulties after the end of their violent relationship, which delayed their PTG. The exact reasons for their poor financial status varied, but they were all direct consequences of the relationship. In some cases, the women described their financial problems as permanent.

*All the money I had was spent during this relationship. When the relationship ended, I had nothing, I was completely bankrupt, financially, psychologically, and physically. I can’t work anymore because I have lost my health. I must live from benefits from now on*

(Marianne)

*As soon as we got divorced, the financial violence started. He took everything away from me. I was always poor after our divorce*

(Heidi)

*The financial violence. I was stuck. He was determined to ruin me financially. He knew what he was doing*

(Wilma)

Some of the women endured other traumatic events after their relationship had ended, such as the loss of their loved one, violence against their loved one, sickness, ruined housing, etc., which caused them sorrow and further difficulties in life. 

**The perpetrator.** A great majority of the women said that their former spouses kept causing them problems after their relationship had ended, which negatively affected their PTG. The men harassed them and behaved in threatening ways to frighten them and sometimes to frighten their children.

*For months he kept on stalking me, using every chance he got to harass me and threaten me. He showed up at my home, threatening me, sometimes he even showed up inside my house. My children were very scared. At the end he attacked me in my workplace*

(Frida)

*Sometimes, when I met him after our divorce, he grabbed me when I was holding our child and started to shake me, with the crying child in my arms. It was terrifying for both of us*

(Kimberly)

This behavior sometimes led to nightmares and flashbacks for the women. They were also afraid of the perpetrators coming back to hurt them or to force themselves back into their lives in a violent way.

*I used to have flashbacks and nightmares about him stalking me. I was really afraid of him at the time*

(Grace)

Some of the women reported that the psychological violence became worse after the end of the relationship, which delayed their PTG. A few participants reported finding it hard to let go of their former spouses, since the men constantly reminded them of their existence, which served as an obstacle on their journey to PTG. A few said they had tried to hold on to the good sides of the men, even feeling obsessed with them.

*When he was not trying to get me back, he was threatening me. He constantly called me, threatening to shoot himself, saying that he had no money because he had to pay me child support, that I had ripped him off, complaining about me having such a nice life*

(Heidi)

*For a few years he was everywhere, sending me messages, telling me how disgusting I was, sending messages to my friends and my new boyfriend. No matter what I did, he was there somehow, harassing me. I was really tired of this behavior, but a part of me had difficulties in letting him go. I didn’t really get the opportunity to let him go and move on with my life*

(Lydia)

*After the end of the relationship and I had been alone for a while, I started to feel like I had been sailing alone in a very turbulent sea with a great storm blowing against me. I desired to find my harbor, just falling down on my knees and relax. I had been working so hard to survive. I still wanted to fall into his arms and collapse there to stop fighting for a while, it felt really tempting. I felt really bad at that point*

(Phoebe)

**The children.** Many participants said they were sad and angry on behalf of their children because of what the children had witnessed and the violence that some of them had to endure, these negative feelings serving as a delay in their PTG. Some women described their feelings of anger because of the perpetrator’s continuing bad behavior towards the children after the abusive relationship had ended.

*I was so angry with him because of his behavior towards the kids through the years. I see it now, after the kids have grown up, that I used a lot of energy in being angry with him on their behalf*

(Yvonne)

*I always regret, that my child had to witness all this. You know, him yelling at me in front of the child. I always regret that*

(Sarah)

*I feel sorry for my children, that they had to endure these violent circumstances because I remember so clearly how I felt, when I was a child, living in a violent home*

(Rachel)

**The law and the institutional social system**. Participants described their negative experiences of the institutional social support system. They reported how regulations and laws made their lives difficult in many ways, leading to challenging circumstances and feelings, which delayed their PTG.

*Of course I am angry about our system, the rules in our society, angry about that one person can decide that he is going to keep all your assets, both assets that you have in common and your personal assets and no matter what you say, he can get away with it!*

(Wilma)

*The system is not welcoming you. I can understand it up to some point, when you are getting a divorce and have a child and all that, but to have to settle things with your perpetrator, sitting there beside him, confronting him, talking to him, trying to find a solution there and describing the violence in your relationship, they just didn’t care*

(Kimberly)

For many participants, it took a long time to end their marriages or cohabitations since the perpetrators did not agree to do so. This could cause the women great problems that negatively affected their lives in various ways.

*It took a very long time to end our cohabitation, which caused me great financial problems. You didn’t have the legal rights as a single mum, such as discount of the kindergarten fees, financial support as a single mum*

(Kimberly)

*Since it took such a long time to end it formally, by law, I couldn’t get the help we needed from the social services. I didn’t want to go there, going down on my knees and whine about the violence to get what I needed for us. I just wanted to end the relationship by law and have my rights like everybody else*

(Heidi)

The majority of the women were forced by law to communicate with the perpetrators due to the children they had with them. They were also forced to send their children to these men, even though they did not trust them, and in some cases even the children were afraid of their father.

*Since we have a child together, I am forced to communicate with him, even though I don’t want to*

(Grace)

*They don’t want to go to their father. I managed to defend my children when we were together, but now they have to meet him without me defending them, every second* *weekend, and his new girlfriend doesn’t want my children, she is not nice to them. I don’t trust these people*

(Phoebe)

*My children are afraid of their father, he has been violent to them after our divorce. And they have to live with him every second week, because he didn’t beat me enough I guess our system is so broken, it has so many flaws*

(Rachel)

Some women reported their fear of the child protection services coming and taking their children away from them. Additionally, some of the men tried to control the women’s lives by using the children as weapons in that battle; threatening to take the children away from them, threatening to start a custody dispute that would last years, etc. These circumstances often caused delay to their PTG.

*I was terrified that Child Protection would take my child away from me, because of him being violent to me in front of the child after our divorce*

(Kimberly)

*He used his lawyer to control me, threatening to take the children away from me if I didn’t agree to what he wanted. I was so scared that he would get the custody of the children, I agreed to everything*

(Heidi)

## 4. Discussion

The purpose of this study was to identify the obstacles to PTG as experienced by female survivors of IPV. The overriding theme of the study was “It was all so confusing”, which expressed the core of participants’ feelings when describing the obstacles on their journey towards PTG. The results demonstrate that the majority of the obstacles that participants met during their PTG were intrapersonal, i.e., their negative personal feelings and their negative perspective towards themselves. Other obstacles reported by the participants were physical and psychological health problems, challenging personal circumstances, the perpetrator, and the law and institutional social systems. Participants reported experiencing feelings of diminished worth in the fields they described as obstacles in their PTG, which influenced their lives in a negative way, setting them back to survival mode that kept them from the phases of recovery and healing and delayed their journey to PTG. This feeling of diminished personal worth was based on their inner experience of themselves and their situation, but it was also based on the negative actions and reactions they endured from their surroundings and the system.

The nature of IPV is complex, intimate, and chronic, and the trauma recovery is unique [21]. Even though the effects of such trauma are well known, the possibility of PTG following IPV is less known, though the literature on PTG is growing. In the results of a recent qualitative study among women who had experienced PTG following IPV, the factors that facilitated their PTG following IPV were described [30], which is very important information when attempting to help female survivors reach PTG. To further promote PTG among women following IPV, it is important to be aware of the obstacles they may meet on their journey towards PTG. When combining these two aspects in building PTG, obstacles and facilitators, it is possible to get a clearer overall picture of what should be encouraged and what one should be aware of when assisting survivors of IPV on their journey towards PTG. 

In a study on the meaning of life among eight women following psychological IPV, the results indicated that the participants possessed less self-kindness, which was related to less positive reframing, less growth, and less of a sense of meaning in life [16], which the findings of this study support. When looking at the perception of PTG among women who have reached PTG following IPV, strength, self-respect, and appreciation of self are three of the sixteen most valuable aspects participants described as part of their PTG [30] (p.15). When adding the results of the current study, the importance of supporting female survivors of IPV in processing their negative personal feelings and their negative perspectives towards themselves should be emphasized when guiding them on their journey to PTG. The results of a recent study of Salvadoran women who had survived IPV revealed a higher prevalence of mental disorders, somatoform disorders, and somatic complaints, along with suicidal thoughts, among them than in those who had not endured IPV [40]. According to the literature in the field of IPV, negative emotions similar to PTSD, such as shame, fear, and guilt, can be influencing factors in maintaining the violent relationship [4], and have negative effects concerning help-seeking [41] and processing the trauma [42], which also reflects the feelings of the participants of this study following their experience of IPV, adding information on how the negative impact of their symptoms following IPV served as obstacles to their PTG. In a study of post-traumatic effects and IPV, the results revealed that even though the victim’s violent partner was absent, or the danger was not real, the terror remained in the woman’s life [42]. These results are supported by the results of this study, with participants describing how their perpetrator kept “hovering around them like a fly”. Leaving a violent partner has been reported in the literature on IPV not only as an important risk factor for deadly violence and injury but also for the health of the woman herself. Women separating from a violent spouse are at great risk of stress and experiencing mental and physical health problems, as well as enduring great conflict over their children, being concerned about their safety. They also tend to have economic, structural, psychological, and social barriers to help-seeking [43]. In another recent study, where women who had suffered IPV described the facilitating factors on their path to PTG following IPV, most of the facilitating factors in their PTG were intrapersonal [30]. The results of this study are parallel to these results, since most of the concepts the participants used to describe the obstacles to their PTG were also intrapersonal. 

The results of this study reveal the broad range of obstacles encountered by women on their journey towards PTG following IPV. These findings emphasize the necessity of considering each woman’s life and circumstances in a holistic way when supporting them on that journey, which requires an interdisciplinary approach. It is significant for the victims of IPV, their loved ones, and professionals supporting them to be aware of those possible obstacles, to keep on going, and not to give up when meeting them on their journey towards PTG. Because of the high international prevalence of IPV, it is useful for communities and authorities to be aware of the obstacles to PTG in order to identify changes that could be made to institutional systems and routines to reduce the obstacles to PTG for female survivors of IPV. 

Considering these findings, there is a reason to review the resources already available for female survivors of IPV to better promote their PTG. This could be accomplished, e.g., by focusing on both intra- and interpersonal factors and their interaction. 

### Limitations of the Study

Participants self-reported their PTG, which may involve a bias in the sample selection. The participants’ willingness and ability to verbally describe their experience of the obstacles on their journey towards PTG could also be a limitation of this study. The authors do not have data regarding the participants’ socioeconomic situation, family status, occupation, or religion, which is a limitation of the study, and the fact that verification by participants was not possible due to circumstances. A further limitation of this study could be that the criteria for participation were that participants had to be able to understand and read Icelandic, which may have led to homogeneity in the sample and to bias in the research results. The large sample size of the study can be seen as a limitation when drawing out the complexities of the phenomenon in detail. Thus, it could be useful in future studies of this phenomenon, to use a smaller sample in order to better elaborate on similarities and differences between participants’ stories.

The aim of the study was to increase the knowledge and deepen the understanding of the phenomenon and not to generalize about the findings.

## 5. Conclusions

The results of this study apply to the field of research on women who have experienced PTG following IPV and help to represent the factors that are likely to be obstacles for women on their path towards PTG. The results address the importance of assisting survivors of IPV in confronting and working their way through the hindrances reported by the participants and can play a valuable role in promoting their growth. To achieve PTG following the experience of IPV is not only valuable for the women themselves but also their children, their loved ones, and their community.

## Figures and Tables

**Table 1:** The working definition of post-traumatic growth (PTG) used in the study ([8], (p. 4)).

An individual who has reached post-traumatic growth experiences positive personal changes as a result of a struggle with a traumatic event. The individual has increased personal strength, improved relationships with others, experiences positive changes in attitudes and appreciation towards life, and sees new possibilities in life. The experience, though negative in itself, has had positive meaning for the person.

**Table 2:** The 12 basic research steps of the Vancouver School process and how they were followed in the present study.

Steps	Description of Steps	What Was Done in This Study
*Step 1.* Selecting dialogue partners *(the sample)*	Efforts are made to select participants who have both typical and nontypical experiences of the phenomenon.	Twenty-two female survivors of IPV, aged 23–56, who self-identified as having reached PTG participated in the study.
*Step 2.* Silence *(before entering a dialogue)*	Preconceived ideas are considered, written down, and deliberately set aside.	The researchers reflected on their preconceived ideas and consciously set them aside as much as possible.
*Step 3.* Participating in a dialogue *(data collection)*	One or two interviews are conducted with each participant. The number of participants is not determined in advance. It is determined by data saturation i.e., how many participants are interviewed and how many interviews are conducted, often 12–18 interviews.	Each participant was interviewed once by the first author, in all 22 interviews because not much is known about the obstacles to PTG. In the interviews, the first author who conducted all the interviews listened reflectively.
*Step 4.* Sharpened awareness of words *(beginning data analysis)*	All interviews are recorded, transcribed verbatim on a computer, and encrypted. Data analysis starts in the interviews and therefore data collection and data analysis run concurrently. After transcribing the interviews, the transcripts are treated as text and the researcher reads the transcripts reflectively.	All the interviews were recorded, transcribed verbatim on a computer, and encrypted. The first author then repeatedly read the transcripts and analyzed them in detail by marking texts and writing comments in the margins, which contributed to answering the research question. Nvivo 12 was also used in the data analysis.
*Step 5.* Beginning consideration of essences *(coding)*	The researcher reads the transcripts again, repeatedly pondering on what is the essence of what this participant is saying together with finding key phrases and their meaning. The researcher then analyzes the text into main themes and subthemes.	Every interview was further analyzed through labeling, categorizing, and organizing the data into main themes and subthemes to begin constructing the essence of the experience.
*Step 6.* Constructing the essential structure of the phenomenon from each case *(individual case construction)*	To understand the overall picture of each individual’s experience, the main themes in each participant’s story are highlighted and the main points are presented in an analytical model for each individual.	The main themes and subthemes in each woman’s story were emphasized and the most significant themes were built into an individual analytic framework.
*Step 7.* Verifying each case construction with the relevant participant *(verification 1)*	Each individual analytic model involves a specific interpretation of the researcher. Each participant is asked to confirm the researcher’s interpretation.	Owing to circumstances, this step was not performed, unfortunately, which is a methodological limitation of the study.
*Step 8.* Constructing the essential structure of the phenomenon from all the cases *(meta-synthesis of all the different case constructions)*	The researcher tries to understand the overall analytic framework of the phenomenon itself, to realize what the participants’ shared experience is and what is different. The researcher constructs an overall analytic framework for all participants.	To construct one main analytic framework, all individual analytic frameworks were compared. It was in this final data analysis that the second author stepped in and the two authors reflected on the data and reconstructed part of the preliminary findings together.
*Step 9.* Comparing the essential structure of the phenomenon with the data for verification *(verification 2)*	The researcher compares the written interviews with the overall analytic model.	For verification, all the interviews were re-read and compared to the final analytic framework.
*Step 10.* Identifying the overriding theme describing the phenomenon *(construction of the main theme)*	The researcher presents the essence of the phenomenon, which is a conclusion about the phenomenon in a nutshell. That becomes the main theme of the study.	The first author constructed the essence of the experience of obstacles on the journey from IPV to PTG: “It was all so confusing”.
*Step 11.* Verifying the essential structure with the research participants *(verification 3)*	The development of a holistic analytic model is always based to some extent on the researcher’s interpretation. This interpretation needs to be confirmed by some participants.	Owing to circumstances, unfortunately, this step was not performed. This is a methodological limitation of the study.
*Step 12.* Writing the findings *(multivoiced reconstruction)*	When writing the results of the study, the researcher uses direct quotations from the participants so that their voices can be heard, which increases the credibility and trustworthiness of the results. This step results in a multivoiced reconstruction.	The participants were quoted directly to increase the credibility and trustworthiness of the findings and conclusions.

**Table 3:** Examples of questions from the interview guide used in this study.

Questions From the Interview Guide
**Main interview question** Did you experience obstacles on your journey to PTG? If yes, please describe these.
***Examples of follow up questions***
Can you identify some of your own personal feelings and state of mind you felt were obstacles to your PTG?
Did you experience that your physical and psychological health in some way were obstacles to your PTG?
Did you experience any other obstacles to PTG?
Is there something you would like to add to what you have already told me?

**Table 4:** Overview of the fourteen main obstacles on the participants’ journey towards PTG following IPV.

	Main Obstacles on the Journey towards PTG Following IPV
Feeling of Shame	Self-blaming thoughts, fear of losing credibility, fear of being stigmatized, prevented from finding appropriate support, feeling of less worth (i.e., all my fault, should have seen this coming, could not ask anyone for help, did not belong anymore).
Suicidal Thoughts	Difficulties of finding purpose in life, burden of responsibility for the violence, feeling of things being better in their absence (i.e., always doing the wrong things, will ruin the child, best to end this).
Broken Self-Identity	Negative attitudes and severe self-criticism. Destructive behavior towards self and body image (i.e., do not deserve anything good, have an appalling body, allowed the violence to happen).
Insecurity	Feeling anxious and insecure. Difficulties in knowing who they were, feeling aimless in life (i.e., similar to free fall, did not realize she could “fly”).
Feeling Alone and Isolated	Isolation and feeling of loneliness leading to increased vulnerability (i.e., no one was left anymore, uncomfortable to be all alone).
Triggers	Triggers related to their experience of IPV that led to bad feelings and had negative effects on wellbeing. Needed to be processed (i.e., yellow car, pregnancy, column in the paper).
Mixed Negative Feelings	Variety of negative feelings that needed to be processed (i.e., grief, anger, regret, fear, vulnerability).
Emotional Connection to Others	Problems with trusting other people, triggers in later romantic relationships, difficulties in starting new romantic relationships (i.e., always waiting for something bad to happen, overreacting to triggers, feeling of not belonging or not being equal to others).
Physical and Psychological Health	Various health problems due to severe, long-term stress, and gaslighting in the violent relationship (i.e., various physical diseases, broken spirit, feeling of fatigue, reduction or loss of working capacity, and hostility towards self).
Personal Circumstances and Social Surroundings	Financial troubles, social isolation, continuing codependency, fear of others judging them and victim-blaming them. Diminished working capacity and other traumatic events (i.e., poverty, lack of security net, troubles in setting boundaries, fear of confiding their feelings to others, loss of routine, loss of a loved one).
The Perpe- trator	Continuing harassment and threats, flashbacks, increased psychological violence, constant reminder. (i.e., stalking, scaring children, complaining, trying to force themselves back into back into their lives).
The Children	Sorrow and anger on behalf of the children because of the violence (i.e., witnessing violence against their mother, previous and current violence against the children themselves, reminders of their own childhood experience of violence).
Law and Institutional Social System	Forced to communicate and settle with the perpetrator regarding assets, children, and divorce. Children obliged to meet their father. Fear of child protection (i.e., took a long time, gave the perpetrator certain powers, participants could not access their assets, did not get the financial and social support they were entitled to, children were often afraid of their father, perpetrator used children and lawyers as weapons in the battle with the woman).

## Data Availability

The data presented in this study are accessible only to the corresponding author. Due to anonymity, ethical, and personal reasons, the data are not publicly available.
